# 基于捕集柱阵列的制备型二维液相色谱纯化烟草中的4个组分

**DOI:** 10.3724/SP.J.1123.2022.08021

**Published:** 2023-03-08

**Authors:** Yunfei SHA, Junwei XIONG, Yulin ZHAI, Baolei WANG, Zhihua ZHONG, Ting FEI, Duxin LI, Da WU

**Affiliations:** 1.上海烟草集团有限责任公司技术中心, 上海 200082; 1. Technology Center, Shanghai Tobacco Group Co., Ltd., Shanghai 200082, China; 2.苏州汇通色谱分离纯化有限公司, 江苏 苏州 215123; 2. Soochow High Tech Chromatography Co., Ltd., Suzhou 215123, China

**Keywords:** 制备型二维液相色谱, 高纯化合物, 烟草, 捕集柱阵列, 中压色谱, preparative two-dimensional liquid chromatography, high-purity compounds, tobacco, trapping column array, medium-pressure chromatography

## Abstract

二维液相色谱(2D-LC)因具有较高的峰容量,在复杂样品的分离分析中获得了广泛的关注。然而,制备型2D-LC以纯化高纯单体为目标,在方法开发和设备构成等方面与分析型2D-LC有较大的不同,目前尚未得到充分的开发,在大规模的制备纯化中应用较少。本文以一套制备液相色谱模块为分离系统,以稀释泵、切换阀和捕集柱阵列为接口,构建了新型的制备型2D-LC系统,旨在规模化纯化多个活性成分。以烟叶中可以用作医药原料的烟碱、绿原酸、芦丁和茄尼醇等组分为目标物,考察了不同类型填料对样品的捕集效率、过载条件下的色谱保留行为等,优化了制备色谱条件。进而利用在线2D-LC系统实现了烟叶提取物的纯化,通过一次运行获得了4个高纯化合物。该系统具有中压色谱纯化成本低、系统在线运行自动化程度高、稳定性好及容易放大等优点。烟叶中活性化学成分的回收利用对促进烟草行业的发展及带动地方农业经济开发具有重大的意义。

## Abstract (en)

Two-dimensional liquid chromatography (2D-LC) has gained increased attention because of its high peak capacity for separating complex samples. However, preparative 2D-LC aimed at isolating compounds is significantly different compared with one-dimensional liquid chromatography (1D-LC) in terms of method development and system configuration; thus, it is less developed than its analytical counterpart. The use of 2D-LC in large-scale product preparation has rarely been reported. Hence, a preparative 2D-LC system was developed in this study. The system was composed of one set of preparative LC modules as a separation system, with a dilution pump, switch valves, and trap column array as the interface, to enable the simultaneous isolation of several compounds. Tobacco was used as a sample, and the developed system was applied to isolate nicotine, chlorogenic acid, rutin, and solanesol. The chromatographic conditions were developed by investigating the trapping efficiency of different types of trap column packings, and chromatographic behaviors under different overload conditions. The four compounds were isolated in one 2D-LC run with high purity. The developed system features low cost because it employs medium-pressure isolation, excellent automation owing to its use of an online column switch, high stability, and capability for large-scale production. The isolation of chemicals from tobacco leaves as pharmaceutical raw materials could aid in the development of the tobacco industry and promote the local agricultural economy.

二维液相色谱(2D-LC)因其较高的峰容量,在复杂样品的分析和鉴定中发挥了重要作用,商品化的分析型2D-LC仪器在天然产物、蛋白质组学、代谢组学以及环境等领域获得了日益广泛的应用^[1,2]^。对于复杂天然产物的分离纯化,由于设备构成复杂、样品溶解度、两维溶剂兼容性等问题的存在,制备型2D-LC发展缓慢^[3]^。

基于多位选择阀和样品环的接口是2D-LC的主要接口形式,Qiu等^[4⇓-6]^构建了多种制备型2D-LC系统,包括反相、反相色谱(RPLC×RPLC)和正相、反相色谱(NPLC×RPLC)等组合模式;朱靖博等^[7]^以两位八通阀和捕集柱阵列为接口构建了反相-反相2D-LC系统,用以纯化木质素;Zhang等^[8]^构建了以两位十通阀和定量环为接口的制备2D-LC系统,以质谱信号为导向进行药物杂质的纯化;Rezadoost等^[9]^构建了两位六通阀和样品环的中心切割2D-LC系统用于纯化紫杉醇;Marlot等^[10]^对由液液色谱(逆流色谱)构建的制备2D-LC技术进行了综述,其中多位选择阀是主要的接口类型。

常规制备型2D-LC由于切换阀和系统设计,多以实验室规模纯化少量物质以进行结构鉴定和药效活性评价为目标,较难以同样的系统设计放大到工业化规模^[11⇓-13]^。因此,本文构建以四位切换阀和捕集柱阵列为接口,通过创新设计的系统切换流路,实现多组分的在线二维制备。该系统具有易于放大到生产规模的优点。

烟草是中国重要的经济作物之一,在烟草的种植和生产加工过程中,产生了大批烟叶废弃物。一方面,烟叶废弃物的传统处理方式对环境造成了一定的压力和污染;另一方面,烟叶中已知含有300多种化合物^[14]^,其中的生物碱、糖类、氨基酸、有机酸等是重要的医药、食品原料。因此,本文以烟叶为样品,对所构建的制备型2D-LC系统进行验证,为烟叶中药效活性物质的高效生产奠定技术基础。

## 1 实验部分

### 1.1 仪器

制备型2D-LC系统为自主研发设备,由3台HT7050A制备型恒流泵、HT7050A紫外可见光检测器、HT7050A收集器(带有10×30试管架)和2支分离柱C_18_ (460 mm×15 mm i.d., 50 μm)构成分离系统,由多个切换阀和4支捕集柱构成2D-LC系统接口,通过自主开发的ht2D-Prep色谱工作站控制仪器在线自动运行。装填了C_18_、WAX和LH-20填料的捕集柱(50 mm×30 mm i.d., 50 μm)、各系统模块及色谱柱均由苏州汇通色谱分离纯化有限公司提供。分析型液相色谱为Waters 2695液相色谱仪。

### 1.2 试剂

无水乙醇(分析纯,永华化学股份有限公司);甲酸、三氟乙酸(TFA)、磷酸二氢铵、磷酸氢二铵(均为分析纯,上海蒂凯姆实业有限公司);烟草粉末(上海烟草集团有限责任公司技术中心)。

### 1.3 色谱条件

第一维液相色谱(^1^D-LC) 分离柱C_18_(460 mm×15 mm i.d., 50 μm);流动相:A为50 mmol/L磷酸铵溶液(pH 6.5), B为乙醇;检测波长:UV 210 nm;柱温:室温;梯度洗脱程序:0~5 min,5%B;5~40 min, 5%B~55%B;40~41 min,55%B;41~50 min,55%B~100%B;50~70 min,100%B;流速10 mL/min;稀释液:0.1% TFA水溶液,流速20 mL/min。

第二维液相色谱(^2^D-LC) 分离柱C_18_(460 mm×15 mm i.d., 50 μm);流动相:A为0.1%甲酸水溶液,B为乙醇;检测波长:UV 210 nm;柱温:室温;梯度洗脱程序:0~5 min,5%B;5~40 min, 5%B~55%B;40~50 min,55%B~100%;50~70 min,100%B;流速10 mL/min。

分析型色谱 C_18_柱(250 mm×4.6 mm i.d., 5 μm);流速1 mL/min;流动相(烟碱、绿原酸和芦丁): A为0.1% TFA水溶液(pH 6.5), B为乙醇;梯度洗脱程序: 0~5 min,5%B;5~25 min, 5%B~55%B;25~35 min,55%B~100%;35~45 min,100%B;洗脱梯度(茄尼醇): 0~5 min,90%B~100%B;5~15 min,100%B。

### 1.4 样品处理方法

取20 g烟草粉末于烧瓶中,加入200 mL甲醇-水(90∶10, v/v)溶液,超声提取2 h后经定性滤纸过滤残渣,旋蒸浓缩至约50 mL,供2D-LC分离纯化。

## 2 结果与讨论

### 2.1 二维制备液相色谱系统的构建

烟草中含有较多化学成分,以烟碱、芦丁、绿原酸和茄尼醇的含量较高^[15]^。在2D-LC系统的构建过程中,根据样品性质,选择合适的色谱分离模式,并考虑同步纯化多个组分的技术特征,开发了基于捕集柱阵列的2D-LC系统,包括两个流动相泵、1个进样泵、1个稀释泵、多个两位四通阀、两个混合器、捕集柱阵列、色谱检测器、第一维制备柱(C1)、第二维制备柱(C2)和其他连接管线组成,系统配置见图1,原型机实物图如图1c所示。

**图1:**
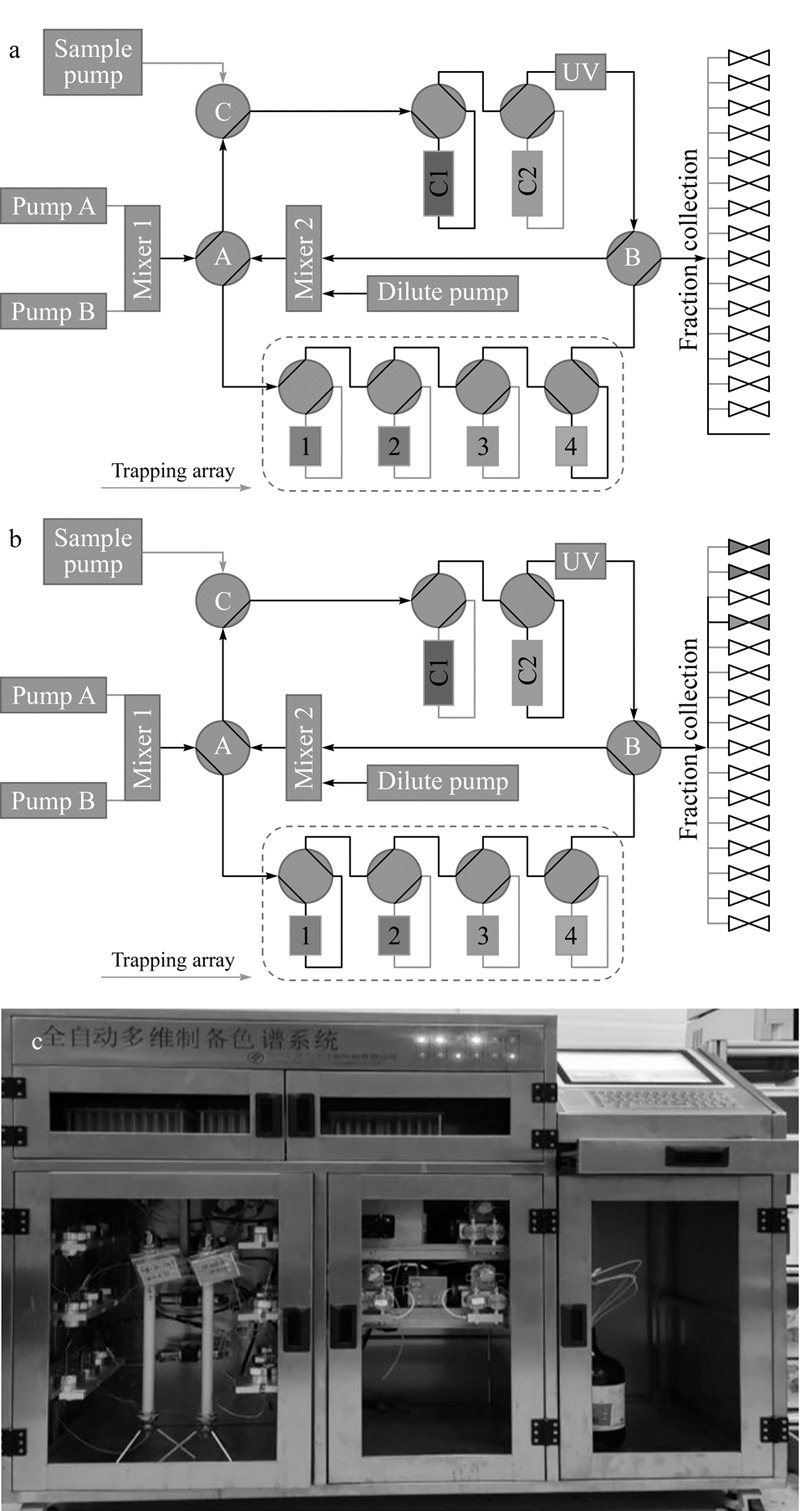
2D-LC系统的结构图

运行^1^D-LC时,流路方向如图1a所示,泵A和泵B输送流动相经混合器混合后,经阀A到达阀C;样品由进样泵输送到阀C,由流动相输送至分离柱1;样品经C1分离后,洗脱液经过阀B到达混合器2,与稀释泵中的溶剂混合后,经过阀A到达捕集柱阵列;通过切换捕集柱阵列中的阀,选择性地将洗脱液导向捕集柱1~4,即分成4个馏分;馏分流经捕集柱,其中的目标组分被捕获,溶剂经过阀B到达馏分收集器,并切换至废液。

运行^2^D-LC时,流路方向如图1b所示,以捕集柱为上样柱,通过切换捕集柱阵列的四通阀选择组分进行第二维分离。泵A和泵B输送流动相在混合器1混合后,经阀A到达捕集柱阵列;切换捕集柱阵列阀,选择捕集柱1为上样柱;流动相输送捕集柱1中的样品,经阀B、阀A、阀C到达C2;捕集柱1中的组分,在分离柱2上分离后,其洗脱液经检测器和阀B到达馏分收集器;馏分收集器根据紫外检测器信号设定的阈值,自动收集馏分。

为了实现规模化生产,系统可靠性至关重要。研制过程中使用了结构相对简单的两位四通阀替代多位阀,各管路连接简单,维护方便;同时,该系统所有液路都有出口,即使出现切换错误也不会导致液路封闭引起局部的超高压、泄露等安全生产危险。

### 2.2 2D-LC分离条件考察

在进行在线2D-LC制备级样品纯化之前,考察各维色谱条件以及载样量是进行纯化工艺开发的重要步骤。首先,考察了二维系统中第一维色谱柱的载样量,如图2所示。烟草提取物样品在上样量为1 g和5 g时,一维色谱图呈现出过载现象(图2a~c),上样量为10 g时出现严重过载现象(图2d),经延长梯度时间至70 min,优化了各组分的分离,烟叶提取物的色谱峰在整个梯度时间内较为均匀,属于可接受的制备色谱过载行为(图3a)。

**图2:**
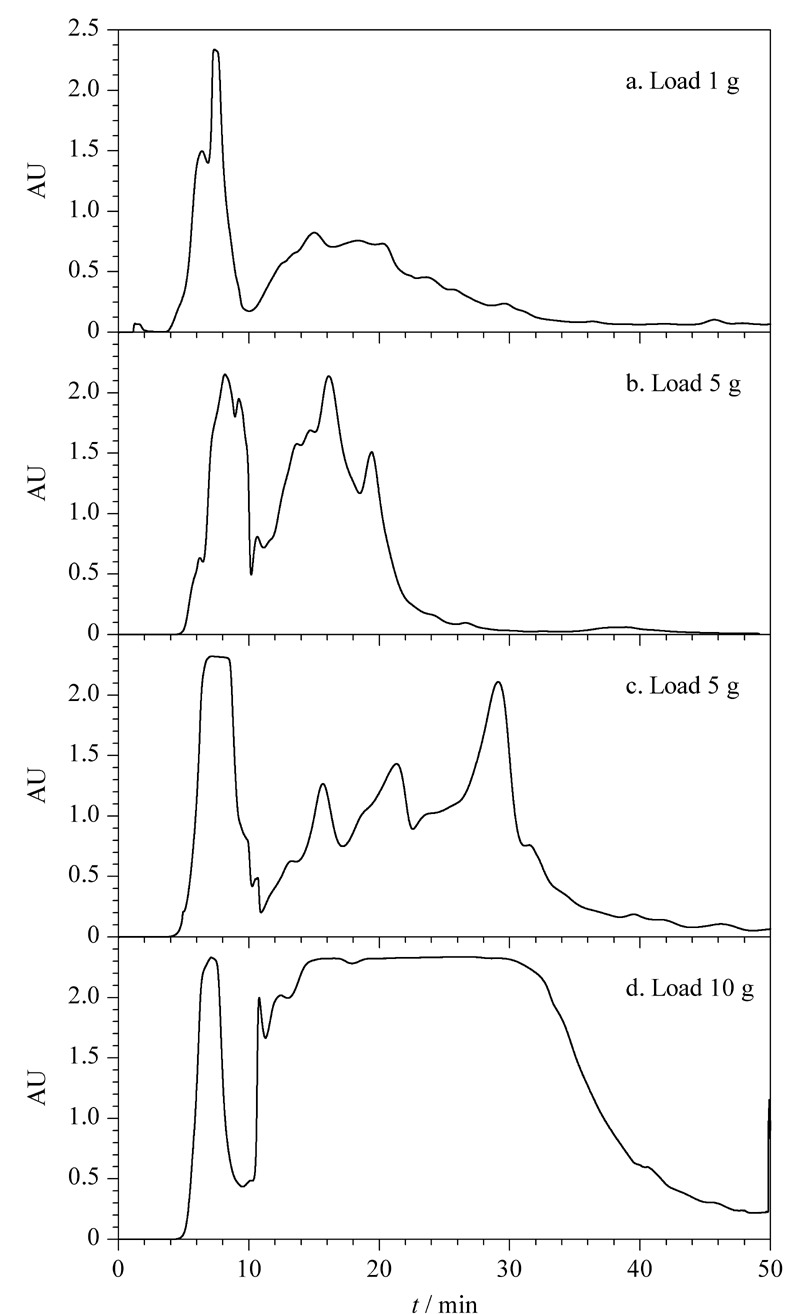
不同载样量及流动相梯度条件下烟草提取物的 一维分离色谱图

**图3:**
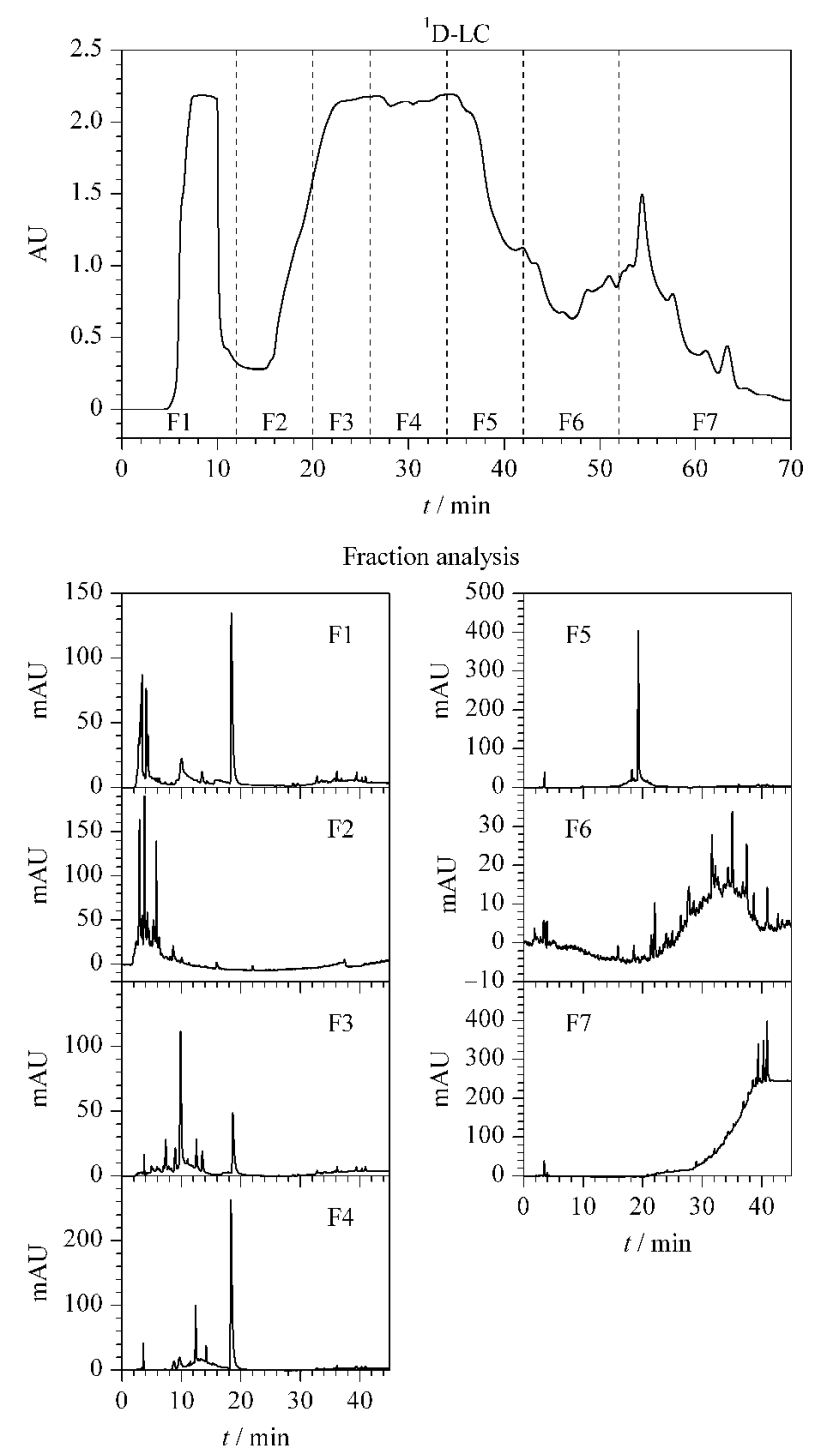
一维液相色谱纯化烟叶提取物及馏分分析色谱图

其次,考察了采用不同类型填料的捕集柱对4种烟叶成分的捕集效果。由于一维馏分中含有有机试剂,导致馏分中的目标组分在捕集柱上无保留或保留较弱,如表1所示。在馏分不稀释的状态下,捕集柱填料为C_18_时,对绿原酸、芦丁和茄尼醇的捕集效率仅为32.70%、38.30%和61.77%;馏分经水稀释5倍后,再上样到捕集柱上,则捕集效率显著提高,分别为56.37%、88.30%和96.13%;馏分经0.1%TFA水溶液稀释,C_18_填料对芦丁和茄尼醇的吸附效率较好;烟碱在不稀释条件下,使用C_18_填料的捕集效率最高。因此,捕集柱1、2、4以C_18_为填料,捕集烟碱组分时不进行稀释,捕集芦丁和茄尼醇时,使用0.1%TFA水溶液稀释;对于绿原酸组分,以WAX为填料时的捕集效率高,但难以洗脱下来;使用LH-20填料时,在不稀释的条件下,捕集效率达到了76.10%,因此,对于绿原酸的捕获,捕集柱填料采用LH-20。

**表1:** 2D-LC接口中捕集柱的捕集效率

Compound	C_18_			WAX (undiluted)	LH-20 (undiluted)
	Undiluted	Diluted with water	Diluted with 0.1% TFA		
Nicotine	65.41	54.02	47.03	10.68	43.97
Chlorogenic acid	32.70	56.37	36.14	100	76.10
Rutin	38.30	88.30	95.67	-	-
Solanesol	61.77	96.13	94.30	-	-

-: not investigated.

最后,考察了4个目标成分的峰分布情况,以确定适宜的捕集柱捕获条件。将第一维分离馏分根据出峰情况手动分成7段(见图3 ^1^D-LC),经分析型LC分析,其色谱图如图3-F1~F7所示,结合标准品进行定性鉴定,确定了馏分1(F1)含有烟碱、馏分3(F3)含有绿原酸、馏分4(F4)含有烟碱、馏分5(F5)含有芦丁、馏分7(F7)含有茄尼醇。F4中的烟碱含量远低于F1组分,因此,舍弃此组分,在线2D-LC制备采用4根捕集柱分别捕获4个组分,其中2号捕集柱填料为LH-20,用以捕获绿原酸,其余3根捕集柱填料为C_18_,分别捕获烟碱、芦丁和茄尼醇。

### 2.3 在线制备2D-LC法分离烟叶成分

制备2D-LC系统中配备了4支捕集柱,在线运行一维液相色谱时,按照目标组分出峰时间区间切换阀,使4根捕集柱分别捕获含有4个目标组分的馏分(见图4 ^1^D-LC),并存储在其中。待第一维运行完成,切换阀模块,依图1b所示流路运行,4支捕集柱捕获的馏分(组分Ⅰ、Ⅱ、Ⅲ、Ⅳ)依次进入第二维进行分离。第二维色谱柱上分离的组分,由馏分收集器收集,采用1.3节分析型色谱进行纯度分析,结果见图4和表2,共有5个组分相对纯度较高(>80%),其中包括一个未知成分。此外,该2D-LC系统还收集到多个其他组分,但纯度较低。在未来有需求时,可以进一步优化二维制备色谱各维的色谱条件,实现更多高纯组分的纯化以及结构鉴定。

**图4:**
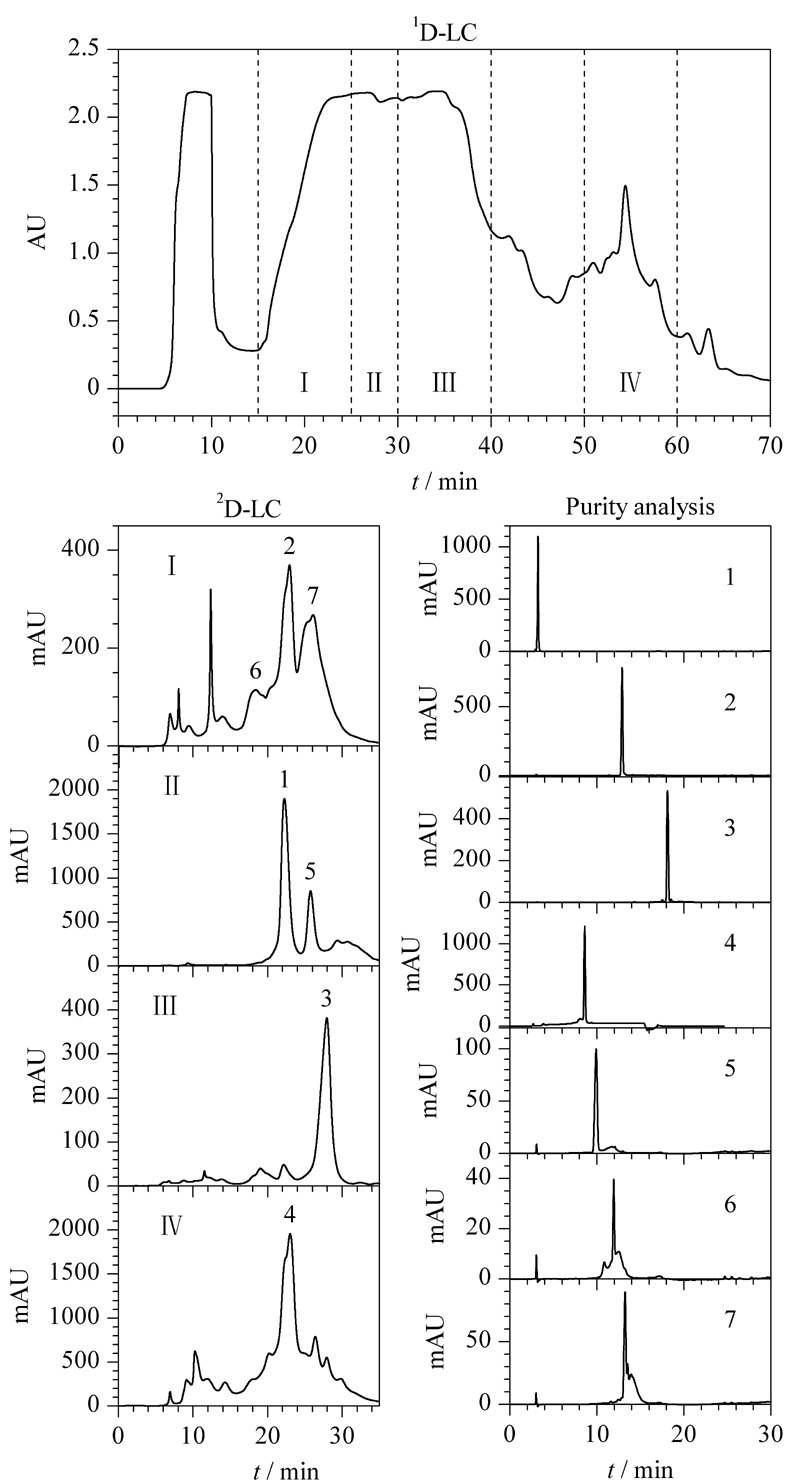
在线制备2D-LC系统纯化烟叶中的活性成分

**表2:** 烟草分离组分的色谱保留时间和纯度

Peak No. in Fig.4	Compound	t_R_/min	Purity/%
1	nicotine	3.23	96.96
2	chlorogenic acid	12.89	98.23
3	rutin	18.12	95.71
4	solanesol	8.59	81.01
5	unknown	9.91	86.83
6	unknown	11.94	41.82
7	unknown	13.24	45.24

通过分离烟叶中的4个主要活性成分,验证了系统简单可靠,且各模块控制及在线监测系统运行正常。经过第二维分离后,获得的烟碱、绿原酸、芦丁的纯度均大于95%,而茄尼醇的纯度大于80%(与市售对照品纯度相当)。以上结果验证了该在线2D-LC系统可以有效地实现目标组分的纯化。

## 3 结论

本文构建了基于捕集柱阵列的在线制备型2D-LC系统,以制备液相色谱模块为分离系统,以稀释泵、切换阀和捕集柱阵列为接口,成功地应用于分离烟草样品中的烟碱、绿原酸、芦丁、茄尼醇等化合物。通过一次上样获得了4个纯度较高的组分,实现了烟叶中活性成分的同步分离纯化。

该系统采用四通阀代替了常规制备2D-LC中的多位选择阀,并进行了创新的接口设计,结合捕集柱阵列模式,能灵活地选择性分离烟草中的目标化合物,实现多个高纯化合物的规模化生产。同时,采用了中压色谱系统,以50 μm粒径填料为分离介质,系统压力低,对设备的要求大大降低,更易实现自动化操作和放大到制备规模,在烟草的快速分离制备和工业化生产方面具有广阔的应用前景。此外,该系统的概念设计和运行模式也可以应用到其他天然产物的分离纯化中,实现复杂成分的高效纯化。
